# Characterisation of the Virome of Tonsils from Conventional Pigs and from Specific Pathogen-Free Pigs

**DOI:** 10.3390/v10070382

**Published:** 2018-07-20

**Authors:** Anne-Lie Blomström, Xingyu Ye, Caroline Fossum, Per Wallgren, Mikael Berg

**Affiliations:** 1 Department of Biomedical Sciences and Veterinary Public Health, Swedish University of Agricultural Sciences, Box 7028, 750 07 Uppsala, Sweden; yexingyu1989@hotmail.com (X.Y.); caroline.fossum@slu.se (C.F.); mikael.berg@slu.se (M.B.); 2 Guangyuan Center for Animal Disease Control and Prevention, Guangyuan 628017, China; 3 National veterinary institute (SVA), 751 89 Uppsala Sweden; per.wallgren@sva.se

**Keywords:** viral metagenomics, pigs, respiratory disease, specific pathogen-free pigs

## Abstract

Porcine respiratory disease is a multifactorial disease that can be influenced by a number of different microorganisms, as well as by non-infectious factors such as the management and environment of the animals. It is generally believed that the interaction between different infectious agents plays an important role in regard to respiratory diseases. Therefore, we used high-throughput sequencing combined with viral metagenomics to characterise the viral community of tonsil samples from pigs coming from a conventional herd with lesions in the respiratory tract at slaughter. In parallel, samples from specific pathogen-free pigs were also analysed. This study showed a variable co-infection rate in the different pigs. The differences were not seen at the group level but in individual pigs. Some viruses such as adenoviruses and certain picornaviruses could be found in most pigs, while others such as different parvoviruses and anelloviruses were only identified in a few pigs. In addition, the complete coding region of porcine parvovirus 7 was obtained, as were the complete genomes of two teschoviruses. The results from this study will aid in elucidating which viruses are circulating in both healthy pigs and in pigs associated with respiratory illness. This knowledge is needed for future investigations into the role of viral-viral interactions in relation to disease development.

## 1. Introduction

Viral infections are of major concern within the pig production industry, in that they cause not only severe disease but also subclinical infections [1,2] that can have severe economic consequences [3,4]. It is becoming evident that a number of factors, including multiple microorganisms, often act synergistically to create a certain clinical picture. This is particularly evident in, for example, complex respiratory and enteric diseases. Known major respiratory viruses include *porcine reproductive and respiratory syndrome virus* (PRRSV), swine *Influenza A virus* (swine AIV), and *pseudorabies virus* (PRV); *porcine respiratory coronavirus* (PRCV) and *porcine circovirus type 2* (PCV2) are also believed to be involved [1,2]. It is highly plausible that other viruses could be of importance as well, but they have been overlooked. Some reasons for this could be that diagnostic labs are not searching actively for them, because they are not believed to be involved in respiratory diseases, or because they have not even been discovered yet. Additionally, it is known that the combination of viruses and/or bacteria, as well as different management and environmental factors, are of importance. A viral infection that under certain circumstances does not cause any apparent problems for the infected host can, under other circumstances, have severe consequences. Considering this, it is important not only to identify individual viruses in a host but also to investigate the entire viral community.

High-throughput sequencing (HTS) combined with metagenomic approaches has been shown to be a powerful tool for elucidating the aetiology behind diseases, as well as identifying novel viral species from humans, animals, and plants [5,6,7]. For pigs, many novel viruses have been identified, including *porcine circovirus type 3* (PCV3) [8], *porcine bocavirus* (PBoV) [9], and *atypical porcine pestivirus* (APPV) [10]. The role that many of these viruses potentially play in disease development is yet to be determined. Apart from detecting previously unrecognised viruses, HTS and metagenomics is a valuable tool for investigating the viral community [5,6,7,11].

In this study, viral metagenomics was used to characterise the viral community of individual pigs coming from a conventional herd with respiratory lesions at slaughter with the aim of identifying possible agents connected with this disease complex. To understand complex diseases and the role that different viruses may or may not play, we need to identify which viruses are circulating in the porcine population regardless of health status. Thereby, we could possibly identify viruses that make up a basal virome at different ages, in different herds, in connection to different health statuses, etc. This knowledge could be used to understand the effects of different viral-coinfections. In pursuit of this goal, we also investigated the virome of specific pathogen-free (SPF) pigs.

## 2. Materials and Methods

### 2.1. Animals

Tonsil samples were obtained from pigs aged 9–11 weeks being used as untreated control animals in a study on a saponin-based adjuvant [12]. The experiment was approved by the Uppsala Ethical Committee on Animal Experiments (Reg. No. C 105214/15, 23rd of October 2015). In brief, eight tonsils from SPF pigs (Yorkshire × Landrace) originating from a herd (Serogrisen, Ransta, Sweden) declared free from major swine pathogens [13] were collected. The viruses the SPF pigs were declared free from included swine AIV, PRRSV, PRV, *porcine epidemic diarrhea virus*, *African swine fever virus*, *Japanese encephalitis virus*, *foot and mouth disease virus*, *rabies virus*, *classical swine fever virus*, *swine vesicular disease virus*, and *transmissible gastroenteritis virus*. Similarly, eight tonsils were obtained from conventionally reared pigs from a farrow-to-finish herd with a high prevalence of respiratory lesions (pneumonia and pleuritic) registered in the respiratory tract of the pigs at slaughter. *Porcine circovirus type 2* (PCV2) was known to be present in both herds, but there were no clinical signs of PCV2-associated diseases. None of the pigs included in the study were vaccinate against influenza, nor any other respiratory pathogen.

### 2.2. Nucleic Acid Extraction

The tonsils were homogenised in sterile PBS using Precellys CK14 tubes (Bertin Technologies, Montigny-le-Bretonneux, France) prior to filtration (0.45 µM) and DNase I (50 U)/RNase (5 µg) treatment. The RNA was then extracted using GeneJET RNA extraction kit (Thermo Fisher, Waltham, MA, USA) and eluted in 40 µL EB. The DNA was extracted using the GeneJET DNA extraction kit (Thermo Fisher, Waltham, MA, USA) and eluted in 50 µL nuclease-free water.

### 2.3. Nucleic Acid Amplification and Sequencing

The DNA was amplified using random PCR as described elsewhere [14]. The PCR tag-sequence was cleaved away using EcoRV (Thermo Fisher, Waltham, MA, USA). The RNA was reverse-transcribed into cDNA and amplified by single-primer isothermal amplification (SPIA) using the Ovation RNA-Seq V2 kit (NuGEN, San Carlos, CA, USA) according to the manufacturer’s instructions. The random PCR products and the SPIA products were then purified using the GeneJET PCR purification kit (Thermo Fisher, Waltham, MA, USA) according to the manufacturer’s instructions. The concentrations of the purified random PCR products ranged from 18 to 25 ng/µL, and those of the purified SPIA products ranged from 36 to 66 ng/µL. All the products were sequenced at SciLifeLab/Genome Center (Uppsala, Sweden) using the Ion S5 XL system (Thermo Fisher, Waltham, MA, USA) and two 530 chips. The main type of sequencing errors associated with this platform are insertions and deletions (indels).

### 2.4. High-Throughput Sequencing (HTS) Data Annotation

The raw HTS data were imported to CLC Genomics Workbench (version 11) (Hilden, Germany) and trimmed based on quality (Q = 20) and length (≥50). De novo assembly using CLC Genomics Workbench was performed to find overlapping reads and create longer contigs. Both the contigs and the remaining reads were then annotated using BLASTx (E-value ≤ 0.0001) in Diamond (version 0.8.26) (https://ab.inf.uni-tuebingen.de/software/diamond/). The Diamond results were visualised using MEGAN (ver. 6.8.20) (http://ab.inf.uni-tuebingen.de/software/megan/). The datasets containing the raw data have been deposited in GenBank under the BioSample accession numbers (SAMN08969257-SAMN08969272).

### 2.5. Viral Genetic Analyses

Certain identified viruses were selected for further genetic characterisation. The selection was mainly based on the number of reads and their distribution across the viral genome. For these viruses, reference genomes were downloaded from GenBank, and CLC Genomics Workbench was used to map back the trimmed reads from the different datasets to the reference genome. Using this method, the coverage of the genomes was obtained, and, in the case of complete coverage, the consensus sequence was extracted. In the case of *porcine parvovirus 7* (PPV7), primers were designed based on the mapped reads to cover the gaps and low coverage regions. The complete PPV7 viral genome was amplified using the KAPA LongRange HotStart PCR Kit (Sigma Aldrich, St. Louis, MO, USA) according to the following protocol: 25 μL reaction mixture of 1× PCR buffer, 1.5 mM MgCl_2_, 0.2 mM dNTP, 0.5 mM of each primer, and 0.625U KAPA LongRange HotStart DNA Polymerase. The reaction was initiated with a 3 min heating step at 95 °C, followed by 35 cycles of 30 s at 95 °C, 30 s at 55 °C and 1 min 20 s at 72 °C, and a final extension step at 72 °C for 2 min. The PCR products were purified with the Thermo Fisher PCR purification kit (Waltham, MA, USA) according to the manufacturer’s instructions and were sent for sequencing at Macrogen. The sequences were assembled using SeqMan (Lasergene 9, DNASTAR, Madison, WI, USA). The phylogenetic analyses were performed using MEGA7 [15]. In summary, the sequences were aligned using ClustalW, and then the evolutionary history was inferred by using the Maximum Likelihood method based on the Tamura Nei model with a bootstrap value of 1000.

## 3. Results

### 3.1. Sequencing Data and Annotation

After the trimming of the sequences to remove short and low-quality reads, approximately 800,000–1,200,000 reads remained from each dataset. These were subsequently used for annotation and downstream analysis. The annotation showed the presence of a large number of vertebrate viruses, including both DNA and RNA viruses, in each individual pig (Table 1). In total, viruses from nine families were identified, and in the majority of these families, not only one but several different genera were recovered. Members of the *Adenoviridae* and *Picornaviridae* family could be detected in all or in nearly all pigs in the study, while other families such as *Circoviridae* and *Caliciviridae* were less common. The reads/contigs annotated to each virus could, in some cases, such as that of teschovirus (see more details below), cover the complete genome, while for others, only a few number of short single reads matched. In the coming sections, a number of the viruses identified will be described more in detail.

### 3.2. Adenovirus

Adenoviruses were identified in all of the investigated pigs. Both *porcine mastadenovirus A* and *B* were present in the population; in some pigs, both A and B were present, and in other cases only one of these was present. The *porcine mastadenovirus A* reads/contigs matched to porcine adenovirus 3, while the *porcine mastadenovirus B* reads/contigs matched to porcine adenovirus 4. There were also several reads/contigs that matched, in the BLASTx search, as a top hit to uncharacterised adenoviruses or to adenoviruses from other species. These have been placed in the “other mastadenovirus” category in Table 1. We did not recover the entire genome from any of the mastadenoviruses identified. However, for several of them, larger mastadenovirus contigs were produced. In one case, a contig of 11,576 base pairs was identified. For *porcine mastadenovirus A*, the trimmed HTS reads, from the dataset (S7) with the most adenovirus sequences identified, was mapped back to the most similar porcine adenovirus 3 genome found in the GenBank (AB026117.1) to see if more of the genome could be recovered. However, even with this approach, it was impossible to recover the complete genome, and only approximately 60% coverage was achieved. However, the reads were spread out across the genome and a 90–100% identity on the nucleotide level and 78–100% identity on the amino acid level were observed. As mentioned, *porcine mastadenovirus B* was also identified in 10 animals, showing high sequence identity to porcine adenovirus 4. However, unfortunately no complete genome sequence was available from GenBank. Only sequences from the *fibre* and the *E1B 55K* genes were available, and compared to these the sequences with Swedish porcine adenovirus 4 displayed a high sequence identity (over 95%).

### 3.3. Porcine Parvovirus 7

**Figure 1:**
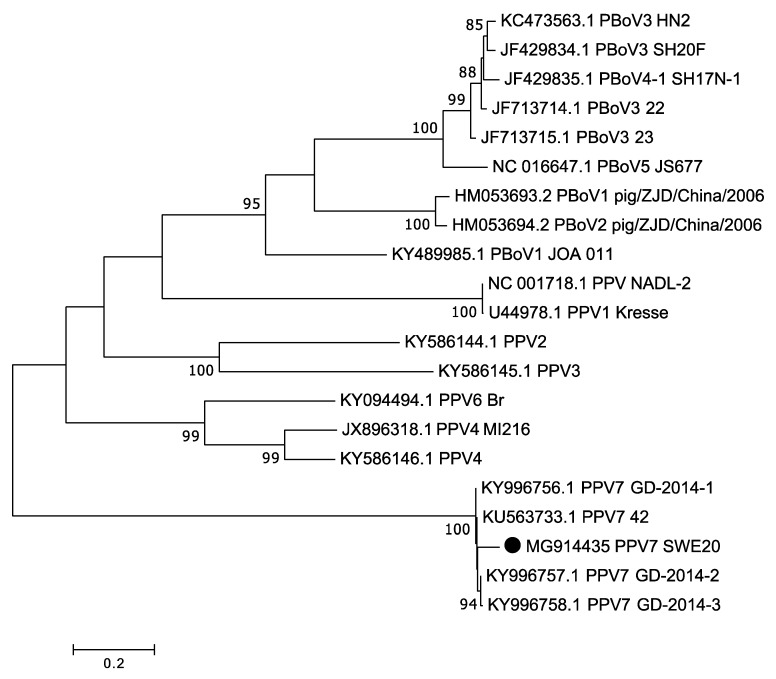
Phylogenetic analysis of porcine parvovirus 7. The evolutionary history was inferred by using the Maximum Likelihood method based on the Tamura Nei model with a bootstrap value of 1000. The analysis is based on the complete nucleotide sequence *NS1* gene, and, in total, there were 1483 nt positions in the final dataset. The PPV7 obtained from this study is marked with a black circle, and only bootstrap values greater than 70% are shown.

One or several members of the *Parvoviridae* family was identified in 12 out of the 16 pigs. We have previously shown that *porcine bocavirus 1* and *3* are present in Sweden [9,14,16]. Therefore, we prioritised to further characterise the PPV7 identified in four of the pigs (two SPF and two conventionally reared pigs), as this virus has not been detected in Europe before, and as many reads were present for this virus in one of the pigs (R4). The metagenomic data from the sample R4 covered 99% of the complete coding region at a sequence depth up to 2480. Together with PCR amplification and sequencing of the missing parts and of low-depth areas, a nucleotide sequence of 4062 bp (accession number MG914435) was obtained. This sequence had two ORFs: one coding for the non-structural protein NS1, and one coding for a putative capsid (Cap) protein. The *NS1* gene was 2019 nucleotides in length, coding for a 673 amino acid long protein. *NS1* from the Swedish PPV7 had a 93.1–94.6% nucleotide sequence identity to the PPV7 *NS1* sequences available from the US and China. The ORF coding for Cap was 1401 nucleotides long, yielding a 467 amino acid long protein. For *Cap*, the nucleotide sequence identity between that of the Swedish PPV7 and those from the US and China was lower (84.7–86.8%) than what was seen for *NS1*. In the 421–552 nucleotide region of the Swedish PPV7 Cap protein, there was high variation compared to the previously characterised PPV7. In this region, both insertions and deletions were observed. The phylogenetic analysis showed that PPV7 from this study grouped with PPV7 from US and China (Figure 1).

### 3.4. Teschovirus

**Figure 2:**
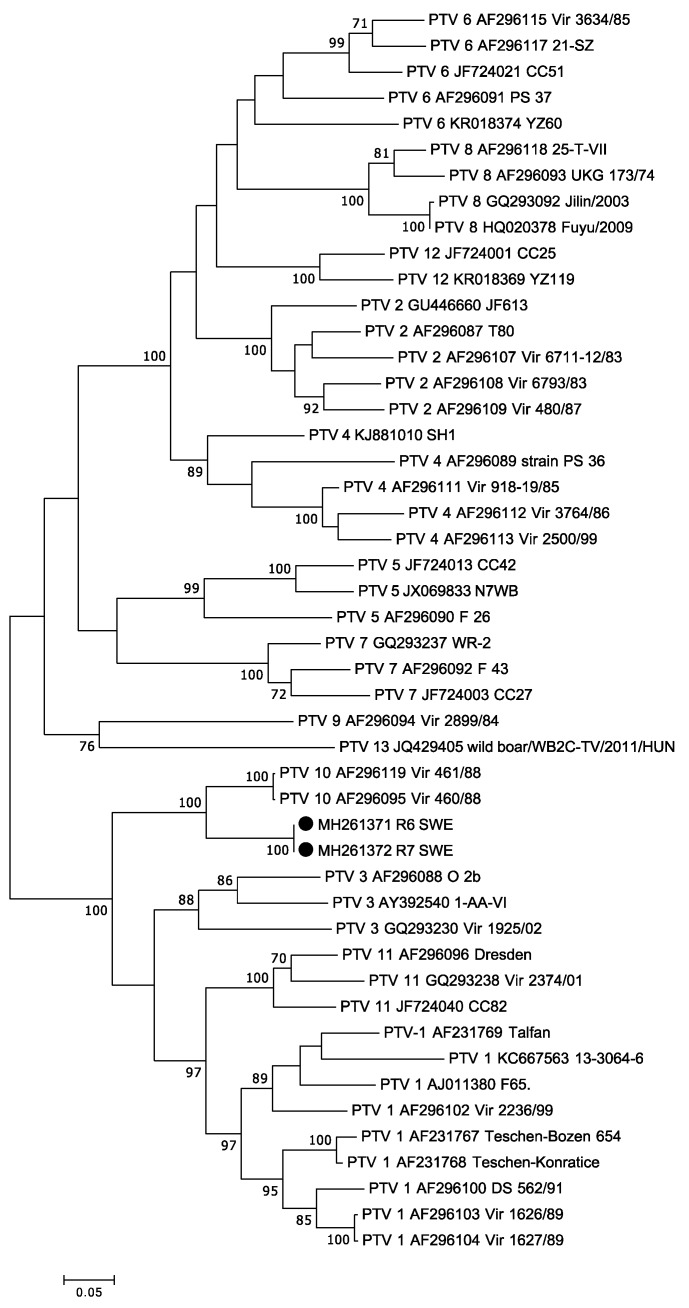
Phylogenetic analysis of teschoviruses. The evolutionary history was inferred by using the Maximum Likelihood method based on the Tamura Nei model with a bootstrap value of 1000. The P1 nucleotide region encoding for the capsid gene was used; in total, there were 765 nt positions in the final dataset. The teschoviruses obtained from this study are marked with black circles, and only bootstrap values greater than 70% are shown.

The majority of the pigs were positive for one or several members of the *Picornavirales* order; these members included *pasivirus A*, *posavirus 1*, and *porcine sapelovirus A*, as well as *porcine teschovirus* (PTV). Due to the high amount of teschoviral reads in some of the samples, *porcine teschovirus* was selected for further genetic analysis. From the two SPF pigs, very few PTV reads were recovered; yet, from two of the conventionally reared pigs (R6 and R7), the complete genome (accession number MH261371-2), including the 5’ and 3’ UTR, was obtained when all the trimmed reads were mapped back to one of the most similar teschoviruses available at GenBank. The coverage/sequence depth for R6 was 6–535 and 2–296 for R7. For both the R6 and R7 teschoviral genomes, one open reading frame (6615 nt) coding for the large polyprotein (2205 amino acids) was identified. Sequence analysis showed that the two Swedish teschovirus genomes had, on the nucleotide level, a 99.9% sequence identity. Comparing them to previously described teschoviruses from other parts of the world, the sequence identity varied between 80.0 and 87.1%. The highest similarity was seen between two teschoviruses belonging to serotype 10; in the phylogenetic analysis, the two Swedish teschoviruses grouped together in the same clade as these teschoviruses (Figure 2).

### 3.5. Other Viruses

Apart from the described viruses, several other viruses were also identified in the tonsil samples (Table 1). Circular viruses belonging to the *Circoviridae* and *Anelloviridae* families were identified in two and six samples, respectively. PCV2 was present in two pigs (one SPF pig and one conventionally reared pig), and these sequences showed high sequence identity (99%) to other PCV2 strains from Europe and the US. *Torque teno sus virus* (TTSuV) was present in more samples, in particular in the SPF pigs, as five out of eight pigs were positive. Other DNA viruses included two different herpesviruses: *Suid herpesvirus 2* and *Porcine lymphotropic herpes virus 1*. In the *Picornavirales* order, three viruses in addition to the previously described teschoviruses were identified: *pasivirus A*, *posavirus 1*, and *sapelovirus A*. The porcine sapeloviruses reads showed the closest similarity to *porcine sapelovirus A* strains from Asia. On the nucleotide level, the reads showed 84–90% identity, and on the protein level 72–100% identity to the most similar virus in GenBank. The longest posavirus contig (1375 nt) found in sample R1 was very similar (almost 100% protein identity) to posaviruses discovered in other parts of the world such as the US and Japan. For the pasivirus sequences, the longest contig (1481 nt) showed the closest similarity (82% nucleotide identity and 89% protein identity) to a German isolate from 2014 (SPaV-A/GER/L00721/2014). Additionally, a few sapovirus and astrovirus reads were identified in the samples.

## 4. Discussion

Respiratory problems are common in pig herds worldwide and can be associated with significant production losses [3,17]. They often have a multifactorial background and can be associated with a number of different factors such as environment, management, production system, animal genetics, etc., in addition to different pathogens. Major viral pathogens associated with respiratory disease are PRRSV, PRV, and swine AIV, which are all known to induce respiratory disease and lesions. Other viruses such as *paramyxovirus* (PMV), *porcine cytomegalovirus* (PCMV), PCV2, PRCV, and TTSuV are considered minor pathogens that could play a role through co-infection or in combination with other outer factors [1,2]. In this study, none of the viruses considered major pathogens were detected, which was partly expected, as Sweden has been declared free from PRRSV and PRV. Viruses of minor relevance (TTSuV1 and PCV2) were identified, but only in one pig (R8). In contrast, five SPF pigs were positive for TTSuV1, one of which was also co-infected with TTSuV2.

In general, there was no major difference regarding viruses detected in the two different groups, but the variation was at the individual level. The viruses that differed between the two groups were *porcine lymphotrophic herpes virus 1*, *adeno-associated virus*, unclassified circovirus, and *porcine sapelovirus A* and *sapovirus*, which were only present in conventionally reared pigs, while TTSuV2 only was detected in one SPF pig. The fact that many of the viruses were found in both groups was not surprising, as many studies have shown a high co-infection rate of several viruses in both healthy pigs and in pigs with different disease complexes [14,18,19]. Many respiratory viruses are also known to be ubiquitous in pig populations. It is possible that viral load could play a role in the development of clinical signs, but also that co-infections could have a synergistic effect. For example, enhanced respiratory disease development has been seen in co-infection situations with PRRSV and viruses such as PRCV [20,21], swine AIV [21], and PCV2 [22,23]. The exact mechanisms behind this are not always clear, but it is known that suppression of the immune system and alteration of cytokine responses can be important [24]. Some viruses can also affect macrophage function [25,26]. Considering that different viruses affect the host in different ways, the order that viruses infect their hosts could be important for particular outcomes, hence making them very complicated to study. Furthermore, bacteria are also known to induce respiratory disease, either alone or through viral-bacterial/bacterial-bacterial interactions [1,2]. In addition, migration of parasites through the lungs may also aggravate signs of respiratory diseases.

Looking at the specific viruses, it was clear that viruses of certain families were well represented in most of the pigs investigated. For example, adenoviruses were present in all pigs, and 16 out of 18 pigs were positive for picornaviruses. Porcine adenovirus is ubiquitous throughout the world; although the virus has been isolated in connection to disease investigations, it is not considered a major pathogen but rather is believed to often result in subclinical infection [27]. There are three species of porcine adenovirus recognised: *porcine mastadenovirus A* (porcine adenovirus 1–3), *porcine mastadenovirus B* (porcine adenovirus 4), and *porcine mastadenovirus C* (porcine adenovirus 5) [28]. Of these, porcine adenovirus 3 and 4 were identified in the different samples from this study. A total of 94% of the pigs were positive for porcine adenovirus 3 and 63% for porcine adenovirus 4. No sequence reads were classified as porcine adenovirus 5. There is a lack of complete genomes of adenoviruses available, making the classification of the reads in this kind of dataset difficult. It is likely, for example, that many of the reads referred to as “other mastadenovirus” in Table 1 could be porcine adenovirus 4 but have been classified as “other mastadenovirus” because only the *fibre*, *pVIII*, *E1B 55K*, and DNA repeat region have been previously sequenced. The dataset may also contain more divergent porcine adenovirus/es that are not classified in any of the present genera.

Different parvoviruses are also known to be present, sometimes at high prevalence, in pigs worldwide. We have previously, using metagenomics, discovered *porcine bocavirus 1* [9,16] in Swedish pigs; PBoV3 has also been shown to be present in Sweden [14]. The pathogenicity of these viruses is not well known, and they have been detected in both healthy pigs and in pigs suffering from both respiratory and enteric diseases [29]. In our study, there was no major difference in the detection rate of these viruses between the two groups. In four of the samples, an additional parvovirus, *porcine parvovirus 7*, was identified. This virus was first detected in 2016 from a rectal swab collected from an adult pig in the US and showed a very low identity to any known parvovirus [30]. The virus in that study was detected in different sample types and at a detection rate of 8.6%. Since that first discovery, this virus has also been identified in China [31]. The non-structural protein of the PPV7 characterised in Sweden showed a high degree of similarity (93.1–94.6%) to the US and China isolates, while the capsid was more divergent, with a nucleotide identity of 84.7–86.8%. The potential role of this virus in connection to disease has yet to be determined.

Picornaviruses were also detected to a high degree in the investigated pigs, and altogether four different picornaviruses were identified: *pasivirus A*, *posavirus 1*, *teschovirus*, and *porcine sapelovirus A*. Picornaviruses can cause subclinical infection, as well as diseases ranging from those with mild symptoms such as fever to more severe diseases [32]. In two of the conventionally reared pigs, a high number of teschoviral reads were identified, enabling the assembly of two complete genomes. Porcine teschovirus is endemic in pigs worldwide, and there are 13 known serotypes [28]. Often the infection is subclinical, but PTV-1 is associated with encephalomyelitis (Teschen disease). PTV2, PTV3, and PTV5 have been associated with a milder version called Talfan disease [32]. The viruses identified in these two pigs showed were most similar to PTV-10, which has not been associated with disease, and whether they belong to the serotype 10 remains to be determined.

In some instances, the number of reads that matched to a specific virus was very low, sometimes less than 10 reads (marked with a lighter green in Table 1). It should be noted that although these low read numbers could be due to low abundance viruses or amplification bias, other reasons such as contamination within the sequencing run cannot be ruled out.

In conclusion, we observed a variable co-infection rate in the individual pigs in the two studied groups. The difference was seen on an individual level rather than on a group level. Thus, no specific virus could explain the respiratory disease of these pigs, but the results obtained provide important information on the viruses circulating in pig populations.

## Figures and Tables

**Table 1:** Viruses identified in the viral metagenomic analysis. The table displays the viruses identified in each of the 16 pigs—eight specific pathogen-free pigs (S1 to S8) and eight conventionally reared pigs from a herd with respiratory problems (R1 to R8). Green means that the pig was positive for the specific virus, and the number indicates how many individual reads mapped to each virus; the viruses with less than 10 reads per sample are marked with a lighter green. * Posavirus 1 has not been assigned to a viral family but is placed within the *Picornavirales* order.

		S1	S2	S3	S4	S5	S6	S7	S8	R1	R2	R3	R4	R5	R6	R7	R8
*Adenoviridae*	*Porcine mastadenovirus A*	24	8	66	50	112	4	945	245	2		124	13	14	98	69	38
	*Porcine mastadenovirus B*	376			2147			6619	1361	14666	7		3615	1191		230	9584
	Other mastadenovirus	609		24,010	3167		3	6561	1073	19478	5		7901	2255		206	8138
*Herpesviridae*	*Suid herpesvirus 2*			4	16		4							50			752
	*Porcine lymphotrophic herpesvirus 1*														30		
*Parvoviridae*	*Porcine bocavirus 1*		4	2	2		2	88	2			27	4		31	1	4
	*Porcine bocavirus 3*				1							1					3
	*Porcine parvovirus 7*			1			2						14131	14			
	*Adeno-associated virus*											2		4	2	16	
	Uncharacterised parvovirus				20							3	3	1	1		
*Circoviridae*	*Porcine circovirus 2*								1								1
	Unclassified circovirus																2
*Anelloviridae*	*Torque teno sus virus 1*		4	1	106			8	16								5
	*Torque teno sus virus 2*								1								
*Picornaviridae*	*Pasivirus A*	4	30	29	31	8	30	46		34		4	1	16	125	5	8
	*Teschovirus*				1	8				6			2	67	5939	3385	85
	*Porcine sapelovirus A*											31	56				
Unassigned *	*Posavirus 1*	1			2					17					2		3
*Caliciviridae*	*Sapovirus*											1					
*Astroviridae*	*Astrovirus*			2		1	1	1		1					1	1	
*Retroviridae*	*Porcine endogenous retrovirus*		80	27	47	87	120	50	80	102	128	93	59	64	88	173	133
